# Intermittent preventive treatment and malaria amongst pregnant women who give birth at the Centre Hospitalier Régional Paul Moukambi de Koula-Moutou in southeastern Gabon

**DOI:** 10.1186/s12936-022-04305-4

**Published:** 2022-11-04

**Authors:** Jean Claude Biteghe-Bi-Essone, Roméo Karl Imboumy-Limoukou, Jean Jordan Ekogha-Ovono, Sydney Maghendji-Nzondo, Pater Noster Sir-Ondo-Enguier, Lydie Sandrine Oyegue, Jean Bernard Lekana-Douki

**Affiliations:** 1 Unit of Evolution, Epidemiology and Parasitic Resistances (UNEEREP), Centre International de Recherches Médicales de Franceville (CIRMF), B.P. 769, Franceville, Gabon; 2 Département d’Epidémiologie Biostatistique et Informatique Médicale, Université des Sciences de la Santé, Libreville, Gabon; 3 Département de Parasitologie-Mycologie, Université des Sciences de la Santé (USS), Libreville, Gabon; 4 Département de Biologie, Université des Sciences et Techniques de Masuku (USTM), Franceville, Gabon

**Keywords:** Intermittent preventive treatment, Malaria, Pregnant women, Southeastern Gabon

## Abstract

**Background:**

Evaluating malaria control strategies for pregnant women is essential. The objective of this study was to determine the factors influencing antenatal care (ANC) visit attendance, complete intermittent preventive treatment with sulfadoxine-pyrimethamine (IPTp-SP) and its impact on the health of pregnant women and their newborn babies living in semi-urban and rural areas of southeastern Gabon.

**Methods:**

This transversal study was performed at the Centre Hospitalier Régional Paul Moukambi de Koula-Moutou (CHRPMK). Information regarding age, frequency of prenatal consultations, obstetric history, use of malaria control measures, use of IPTp-SP, malaria diagnostic of women and their newborns, were collected: (i): from birth registers from 1 January, 2018 to 31 December, 2019 and, (ii): a questionnaire from January to April 2020.

**Results:**

In total, 1,851 and 323 pregnant women were included during the first and the second sub-set of study, respectively. In the first sub-set of data, the mean age was 26.18 ± 7.02 years and 96.54% (1,787/1,851) of pregnant women had attended ANC service but 54.45% had complete ANC visit attendance (at least 4 ANC). The complete ANC visit was linked with age (p < 0.001) and profession (p < 0.001). The complete IPTp-SP (at least 3 doses) was 58.87%. Complete IPTp-SP was linked to profession (aOR = 1.49, 95% CI [1.04–2.18], p < 0.001), ANC visit (aOR = 0.176, 95% CI [0.14–0.22], p < 0.034) and age (p = 0.03). Birth weight was higher for babies whose mothers had received complete IPTp-SP (p < 0,001) but the Apgar score was not influenced by the use of IPTp-SP (p = 0.71). In the second sub-set of data, the prevalence of plasmodial infection was 3.10% (95% IC [1.21–5]) and *Plasmodium falciparum* was responsible for 100% of infections. The prevalence of plasmodial infection was the same for all age groups (p = 0.69), gravidity (p = 0.13) and domestic control measures (p > 0.05). A low birth weight was statistically linked to the mother’s plasmodial infection (p < 0.01). Furthermore, plasmodial infection was statistically linked to premature birth (p < 0.001).

**Conclusions:**

It was observed that attendance of women to ANC service and a complete IPTp-SP course is insufficient.

## Background

Malaria is a public health issue and approximately half of the population in the world is exposed. Malaria is particularly severe in tropical areas where *Plasmodium falciparum* is found [1]. Plasmodial infection essentially results in severe and sometimes lethal febrile symptoms [2]. Pregnant women are one of the most vulnerable populations because of their compromised immune system due to pregnancy. In order to fight malaria in pregnant women, the World Health Organization (WHO) recommends a threefold approach: vector control through the use of long-lasting insecticidal nets (LLINs), the use of intermittent preventive treatment with sulfadoxine-pyrimethamine (IPTp-SP), which has a protective effect in the mother and fetus, and finally, early diagnostic of all suspect cases in order to initiate care [3]. These strategies, that have been recommended by WHO [4, 5], have been adopted by all African malaria-endemic countries, and should be delivered through collaboration between reproductive health systems and malaria control programmes, during at least four target antenatal care (ANC) visits throughout pregnancy. Among pregnant women in sub-Saharan Africa, poor antenatal attendance (and concomitantly intermittent preventive therapy) is associated with delivery of low birth weight babies and more newborn deaths. These effects appear to be greater in primigravidae [5]. Preventive therapy with three or more doses of sulfadoxine-pyrimethamine (SP) was associated with higher average birth weight in infants and lower risk of low birth weight than the 3-dose recommended by WHO [6, 7]. Low birth weight (LBW < 2500 g) were associated with increased odds of low fifth minute Apgar score [8] and study has shown that the proportion of low Apgar score was less in pregnant women with complete IPTp-SP attendance [9].

With a birth rate of 3.26 per woman, the health system in Gabon (central African country) is poorly developed. However, there is an improvement in healthcare provider performance, including timing of each IPTp dose for pregnant women [10]. With an average prevalence of 30%, malaria transmission is perennial in Gabon due to its warm and humid equatorial climate, which favours the proliferation of mosquitoes [11]. In order to fight malaria in pregnancy, the country has adopted the 2-IPTp-SP dose, recommended by WHO in 2003. These measures have led to a significant decrease in infection, especially in the most vulnerable (children aged under 5 years old and pregnant women) [12, 13]. In 2012, at least 3-IPTp-SP doses were recommended by WHO. These strategies have been adopted by Gabonese Ministry of Health [10] and attendance to IPTp-SP was associated with a decrease of plasmodial infection in pregnant women, and with the prevention of premature birth and low birth weight [14]. Malaria prevalence in the capital Libreville and its surrounding areas was 6.7 and 5.3% in peripheral and placental blood, respectively [15]. In order to improve women and child health, since 2017 the Gabonese State, through the National Health Insurance Fund (NHIF), provide free ANC visit (antenatal check-up, IPTp-SP course, LLINs), childbirth and post-delivery care in all public health structures for all pregnant women with NHIF. Free childbirth and post-delivery care is dependent on presentation of three ANC visit certificates delivered each quarter during the nine ANC recommended by the Gabonese Ministry of Health. Subscription to the insurance policy is free for the entire Gabonese population.

To evaluate the implemented programmes, a recent study carried out in 2020 in Fougamou [16], a semi-urban area in central Gabon, showed that 94.4 and 47.9% of pregnant women received one and at least three doses of IPTp-SP, respectively, and that the prevalence of *P. falciparum* infection was 11.7%. In the same study, Fleuramie et al. showed that prevalence of plasmodial infection was the same whatever the number of doses of IPTp-SP received by pregnant women. However, no data are available for semi-urban and rural areas in southeastern Gabon [17].

The objective of this study is to determine ANC visit attendance, adherence to IPTp-SP and its impact on the health of newborn babies in semi-urban and rural areas in southeastern Gabon.

## Methods

### Study site and patients

This study was conducted at the maternity ward of the *Centre Hospitalier Régional Paul Moukambi de Koula-Moutou* (CHRPMK) in Koulamoutou (in Ogooué Lolo Province, Gabon), a semi-urban area in southeastern Gabon with a population of 30,643 people. CHRPMK is the reference health structure for the province. This cross-sectional descriptive study involved all pregnant women who gave birth at the hospital.

### Sampling

Socio-demographic information, maternal and gestational ages at delivery, the regimen of intermittent preventive treatment, gravidity and parity (obstetric characteristics) of all pregnant women, as well as the birth weight and Apgar score of their newborn babies were collected: (i) from birth registries dated from 1 January, 2018 to 31 December, 2019; (ii) with a questionnaire from 1 January to 30 April, 2020, filled and signed by the investigator and the woman or parent/legal guardian for minors, after informed consent. Additional information such as knowledge of pregnant women of malaria, presence or absence of window screens in their homes, use of insecticide, ventilation means, and the use of LLINs were collected via questionnaires. The mosquito net was considered treated with insecticide when it was obtained fewer than 6 months ago. Pregnant women with missing data and who declined to sign informed consent were not included, respectively, during the first (1 January, 2018 to 31 December, 2019) and the second (1 January to 30 April, 2020) sub-set of data collection.

Malaria diagnosis was performed for all pregnant women who came to the hospital from 1 January to 30 April, 2020, using the rapid diagnosis test One Step Malaria Ag. pLDH/HPR2 Combo, following the manufacturer’s instructions. All results were confirmed by a thick blood smear observed under light microscopy by: (i) an microscopist at the biomedical laboratory of the CHRPMK by the Lambaréné method [18]; and, (ii) a second microscopist from Interdisciplinary Centre for Medical Research of Franceville (CIRMF). Thick blood smears were defined as positive if any asexual forms of *Plasmodium sp* were observed. The parasite load was determined and expressed as the number of parasites *(*asexual forms) per ul of blood.

Pregnant women were categorized as primi and multigravida for the gravidity and as primi and multipara for the parity depending on the number of self-reported previous pregnancies. In accordance with WHO recommendations, these were considered as ‘low’ and ‘complete’ ANC visit attendance if pregnant women had consulted ‘fewer than four times’ and ‘at least four times’, respectively, during pregnancy. As the WHO recommends at least three doses of SP, the guideline of three doses was used for the current study to classify the variable into two: (i) incomplete/low (0–2 doses); and, (ii) complete (3 or more doses). ‘Coverage of IPTp-SP’ and ‘coverage of ANC visit’ was defined as proportion of pregnant women who received at least one dose of IPTp-SP and who attended ANC at least once, respectively. Neonatal birth weight are measured with very precise digital scales a few minutes after birth and was dichotomized in low (< 2500 g) and normal (≥ 2500 g) birth weights [19, 20]. Apgar score was performed on newborn at 1 min and 5 min after birth, as described elsewhere [21]. Scores ranged from 0 to 10, with higher scores indicating a better physical condition of the newborn.

### Statistical analyses

Data of pregnant women were recorded in Excel 2013 spreadsheets. All pregnant women with missing data were excluded for the analysis. Statistical analysis was performed using the Epi-Info 6 and R version 4.0.5 (2021-03-31) software. Qualitative variables have been described by proportion and quantitative variables by mean, standard deviation (SD), median with inter-quartile range (IQR). The proportions of qualitative variables were compared using the non-parametric Chi-square test or Fisher’s exact test for numbers below 5. Binary logistic regression model was used to compute the adjusted odds ratios (aOR) of independent risk factors. Crude (OR) are presented, the confidence interval was set at 95% (95% CI). Statistical significance was set at α = 5%.

## Results

### Sampling description

**Fig. 1:**
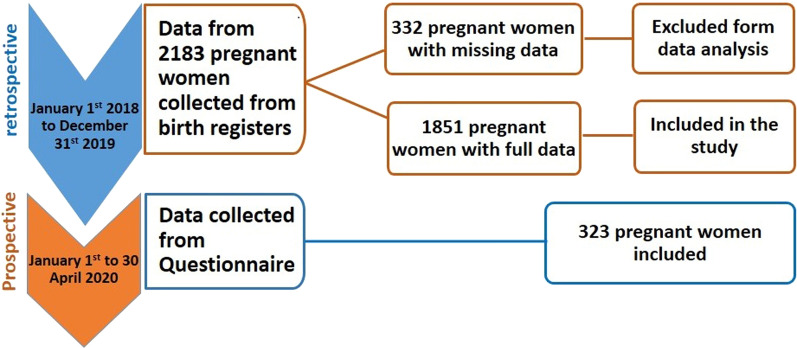
Flow chart of enrolment for pregnant women in the retrospective study

Between January 2018 and December 2019 (first sub-set study), the data of 2,183 pregnant women were collected from birth registries and 332 of them had missing data. Pregnant women with full data included in the analysis are shown in Fig. 1. During the second sub-set study carry out from January to April 2020, data from 323 pregnant women were collected to analyse malaria prevalence, individual factors and relationship between *Plasmodium* infection and birth weight.

In total, data of 1,851 pregnant women were analysed during the first sub-set study. Table 1 shows the demographic characteristics of women. Pregnant women in the 13 to17 and ≥ 18 age groups represented 7% (130/1,851) and 93% (1,721/1,851) of the study population, respectively .

**Table 1:** Demographic characteristics and individual factors of pregnant women January 2018 to December 2019

General characteristics	
*N* (number)	1851
Age	
Mean age (SD)	26.18 (7.02)
Median [IQR]	25.0 [20–31]
Age groups stratified	
[13–17], n (%)	130 (7.02)
[18–22], n (%)	585 (31.60)
[23–27], n (%)	409 (22.10)
≥ 28, n (%)	727 (39.28)
NHIF	
Yes, n (%)	1610 (86.98)
No, n (%)	241 (13.02)
Income-generating activities	
Yes, n (%)	192 (10.37)
No, n (%)	1659 (89.63)
Residence	
Semi-urban regions, n (%)	1804 (97.46)
Rural, n (%)	47 (2.54)
Gravidity	
Primigravida, n (%)	418 (22.58)
Multigravida, n (%)	1433 (77.42)
Parity	
Primipara, n (%)	463 (25.01)
Multipara, n (%)	1388 (74.99)
Gestation period, week (SD)	38.4 (2.04)

**Table 2:** Relationship between ANC visit attendance and individual factors

	*n*	Complete ANC visit n (%)	*p*-value
Age group			
[13–17]	130	53 (40.77)	** < 0.001**
[18–22]	585	306 (52.31)	
[23–27]	409	228 (55.75)	
≥ 28	727	421 (57.91)	
Profession			
Occupation	192	129 (67.19)	** < 0.001**
Unemployed	1,659	879 (52.98)	
NHIF			
Yes	1,610	882 (54.78)	0.81
No	241	126 (52.28)	
Parity			
Primipara	463	266 (57.45)	0.14
Multipara	1,388	742 (53.46)	
Gravidity			
Primigravida	418	237 (56.70)	0.30
Multigravida	1433	771 (53.80)	
Residence			
Semi-urban	1,804	985 (54.57)	0.44
Rural	47	23 (48.93)	

### Antenatal care visit and IPTp-SP chemoprevention January 2018 to December 2019

Pregnant women attended on average 3.60 ± 1.46 ANC visit with a median of four ANC (IQ = [3–5]), a minimum of 0 and a maximum of 8. In total, 96.54% (1,787/1,851) of pregnant women attended ANC service (the coverage proportion of ANC visit attendance). Complete ANC visit attendance was observed in 54.45% (1,008/1,851) of pregnant women. In 13 to 17 and  ≥ 18 age groups, ANC visit attendance was 40.77% (53/130) and 55.49% (955/1,721), respectively, (p < 0.01) Table 2.

In total, 89.52% (1,657/1,851) of pregnant women had received at least one dose (global proportion of IPTp-SP). A complete IPTp-SP course was observed in 57.53% (1,065/1,851, 95% IC [55.27–59.77]) of pregnant women; it increased with the number of ANC visits attended (r = 0.55 [0.51–0.58], *p* < 0.001). Relationships between IPTp-SP, ANC visit and individual factor are shown in Table 3.

**Table 3:** Relationship between IPTp-SP course and individual factors

	*n*	Complete IPTp-SP n (%)	*p*-value
ANC visit			
Complete	1,008	767 (76.09)	** < 0.001**
Incomplete	786	298 (37.91)	
NHIF			
Yes	1,610	929(57.70)	0.99
No	241	139(57.68)	
Age group (in years)			
[13–17]	130	57 (43.85)	** < 0.001**
[18–22]	585	319 (54.53)	
[23–27]	409	228 (55.75)	
≥ 28	727	461 (63.41)	
Profession			
Occupation	192	138 (71.88)	** < 0.001**
Unemployed	1,659	927 (55.88)	
Parity			
Primipara	463	271 (58.53)	0.62
Multipara	1,388	794 (57.20)	
Gravidity			
Primigravida	418	241 (57.66)	0.96
Multigravida	1,433	824 (57.50)	
Residence			
Semi-urban	1,804	1037 (57.48)	0.77
Rural	47	28 (59.57)	

Also, 31.98% (592/1,851) of women had received one or two doses of IPTp-SP (incomplete IPTp-SP); this concerned 33.61% (199/592) of women with complete ANC visit attendance and 65.71% (389/592) of those with low attendance (1, 2 or 3 ANC visits). Nevertheless, 4.17% (42/1,008) of pregnant women with complete ANC visit attendance received no dose of IPTp-SP. Factors associated with complete IPTp-SP are shown in Table 4.

**Table 4:** Binary logistic regression: factors associated with complete IPTp-SP

	n	Complete IPTp-SP n (%)	OR* (95% CI)	aOR** (95% CI	*p*-value
Age group					
[13–17]	130	57 (43.85)	1	1	Ref.
[18–22]	585	319 (54.53)	1.54 (1.03–2.29)	1.32 (0.87–2.02)	0.19
[23–27]	409	228 (55.75)	1.61 (1.06–2.45)	1.29 (0.83–2.00)	0.25
≥ 28	727	461 (63.41)	2.22 (1.50–3.30)	1.71 (1.13–2.61)	**0.01**
Profession					
unemployed	1,659	927 (55.88)	1	1.00	Ref.
Occupation	192	138 (71.88)	2.02 (1.43–2.84)	1.49 (1.04–2.18)	** < 0.001**
ANC visit					
Complete	1,008	767 (76.09)	1	1.00	Ref.
Low	843	298 (35.35)	0.17 (0.14–0.21)	0.18 (0.14–0.22)	**0.03**

OR* (odds ratio); aOR** (adjusted odds ratio and *p*-value, calculated by the binary logistic regression model)

### Characteristics of newborns in January 2018 to December 2019

From January 2018 to December 2019, 1,905 children were born (54 twin pregnancy), 966 males and 939 females with a gender ratio (M/F) of 1.02. More than 80% of newborns had an excellent Apgar score at birth (Table 5), and respectively, three and 29 babies suffered from birth asphyxia (very low Apgar score, between 2 and 3) and stillbirths (very low Apgar score = 0).

**Table 5:** Birth weight and Apgar score of newborns

Parameters	
*N* (number)	1905
Birth weight	
Mean (SD)	2,998.22 (504.49)
Median [IQR]	3,015 [2,710–3,310]
Birth weight groups	
Low, n (%)	254 (13.33)
Normal, n (%)	1,651 (86.66)
Apgar score	
Excellent, n (%)	1,648 (86.51)
Good, n (%)	161 (8.45)
Low, n (%)	64 (3.36)
Very low, n (%)	32 (1.68)

### Number of IPTp-SP doses and impact on newborns January 2018 to December 2019

In total, 1,091 babies were born to women with complete IPTp-SP. The birth weight significantly evolved depending on the number of IPTp-SP doses received by the mother. More pregnant women with incomplete IPT-SP had babies with low birth weight (*p* < 0.001), Table 6. No statistical link was found between the number of IPTp-SP doses and the Apgar score of newborns (*p* = 0.71). However, low birth weight had been associated with very low Apgar score (p < 0.001) (Table 6).

**Table 6:** Binary logistic regression: factors associated with low birth weight in pregnant women

	n	LBW*. n (%)	OR (95% CI)	aOR (95% CI)	*p*-value
IPTp-SP					
Complete	1,091	118 (10.82)	1	1	Ref.
Incomplete	814	136 (16.71)	1.65 (1.26–2.18)	1.67 (1.25–2.24)	** < 0.001**
Parity					
Multipara	1,388	135 (9.73)	1	1	
Primipara	463	89 (19.22)	2.21 (1.63–2.99)	1.79 (1.23–2.59)	** < 0.001**
Age group					
≥ 28	727	67 (9.22)	1	1	Ref.
[18–22]	585	92 (15.73)	1.84 (1.30–2.61)	1.36 (0.92 -2.01)	0.12
[23–27]	409	37 (9.05)	0.98 (0.63–1.52)	0.88 (0.57–1.34)	0.54
[13–17]	130	28 (21.54)	2.27 (1.61–4.52)	1.49 (0.81–2.67)	0.19
Apgar score					
Excellent	1,648	197 (11.95)	1	1	Ref.
Good	161	30 (18.63)	1.69 (1.08–2.62)	1.55 (0.95–2.43)	0.069
Low	64	18 (28.13)	2.88 (1.57–5.23)	2.59 (1.33–4.78)	** < 0.01**
Very low	32	9 (28.13)	2.88 (1.22–6.65)	3.90 (1.65–8.52)	** < 0.001**

LBW* (low birth weight)

### Plasmodial infection in pregnant women from second sub-set data

Only the 323 pregnant women who attended the maternity service from 1 January to 30 April, 2020 benefitted from a malaria diagnosis from peripheral blood at delivery. The mean age was 26.6 ± 6.76 years. The prevalence of plasmodial infection was 3.10% (10/323, 95% CI [1.21–5]). Mean parasitaemia was 817 ± 785 parasites per µl. *Plasmodium falciparum* was responsible for 100% of infections. The average haemoglobin level was 10.3 ± 1.32 g/dL. A complete IPTp-SP course was observed in 66.56% (215/323, 95% IC [61.25–71.49]).

No statistical link was found between the different individual factors, behaviour sand plasmodial infection in pregnant women (Table 7).

**Table 7:** Impact of individual factors and knowledge attitude and practice on plasmodial infection in pregnant women

	n	Plasmodial infection (n = 10)	OR (95% CI*)*	*p*-value
Knowledge of malaria				
Yes, n (%)	320	10 (3.13)	-	0.91
No, n (%)	3	0 (0)		
Window screens				
Yes, n (%)	71	2 (2.82)	0.88 (0.09–4.57)	1
No, n (%)	252	8 (3.17)		
Use of LLINs				
Yes, n (%)	88	2 (2.27)	0.66 (0.09–3.44)	0.73
No, n (%)	235	8 (3.40)		
Ventilation means				
Yes, n (%)	252	7 (2.78)	1.51 (0.31–6.86)	0.46
No, n (%)	71	3 (4.23)		
Use of insecticide				
Yes, n (%)	20	0 (0)	-	1
No	303	10 (3.30)		
Age group (in years)				
[12–17], n (%)	15	1 (6.67)	-	0.69
[18–22], n (%)	97	3 (3.10)		
[23–27], n (%)	69	2 (2.90)		
≥ 28, n (%)	142	4 (2.82)		
Gravidity				
- Primigravida, n (%)	81	5 (6.17)	3.12 (0.76–12.83	0.13
- Multigravida, n (%)	242	5 (2.07)		
Parity				
Primipara, n (%)	85	5 (5.88)	2.91 (0.71–11.97)	0.14
Multipara, n (%)	238	5 (2.10)		
IPTp-SP course				
Complete	215	4 (1.19)	3.10 (0.76–13.42)	0.09
Incomplete	108	6 (5.56)		

### Plasmodial infection and impact on newborns from second sub-set study

No statistical association was found between complete IPTp-SP and *P. falciparum* infection (p = 0.09). The mothers of 12% (6/12) of low birth weight babies were infected by *P. falciparum* (p < 0.001) and *P. falciparum* infection was statistically linked to premature births (p < 0.001), Table 8.

**Table 8:** Birth weight and term of pregnancy according to malaria diagnosis

	n	Plasmodial infection n (%)	OR (95% CI)	*p*-value
Birth weight				
LBW	50	6 (12.00)	9.07 (2.06–45.49)	** < 0.001**
Normal	273	4 (1.47)		
Term of pregnancy				
Pre-term	46	9 (19.56)	65.65 (8.68–2,899.94)	** < 0.001**
Term	277	1 (00.37)		

## Discussion

Pregnant women are one of the most vulnerable populations to malaria, and are more susceptible to plasmodial infections even though these are often asymptomatic [22]. This susceptibility is in part due to the depression of the immune system during pregnancy which allows women to tolerate the fetus [23]. The effectiveness of IPTp-SP has been demonstrated in sub-Saharan Africa [24–26] and in a few localities in Gabon [12, 14]. However, no data regarding semi-urban and rural regions in southeastern Gabon have been published to date. The objective of this study was, on the one hand, to evaluate the level of attendance to prenatal counselling services, and on the other, to assess the adherence rate of pregnant women to IPTp-SP and its impact on newborns, in rural and semi-urban areas of southeastern Gabon.

The mean age of pregnant women was the same as found in urban regions in Gabon (Libreville, the capital, and its surroundings) [10]. Moreover, the same trends were observed in several countries of sub-Saharan Africa, such as Benin, Burkina-Faso and Senegal [24, 25, 27].

One of the objectives of NHIF policy for pregnant women is to improve complete ANC visit attendance and consequently increase complete IPTp-SP courses. The high coverage of health insurance did not result in a high level of complete ANC attendance and consequently of IPTp-SP. Complete ANC visit attendance is significantly lower than that observed in Libreville and its surroundings (Gabon) in 2011 [10] but higher than other countries in Africa, such as Kenya [28] and Benin [29]. These results show a necessity to improve monitoring of pregnant women in southeastern Gabon, probably by multiple awareness campaigns and better monitoring of the criteria for free childbirth (for any woman attending ANC in a health structure recognized by NHIF). More than 85% of pregnant women had NHIF and health workers (midwives) interviewed confirmed that all pregnant women with NHIF had free childbirth. However, it is unknown whether these women had three certificates of complete ANC visit attendance. Despite good health insurance coverage, the reasons of low ANC visit attendance with its consequences for complete IPTp-SP remain to be investigated.

The timing of IPTp-SP delivery complied closely with national guidelines, which stipulate the first dose at 16 weeks gestation. SP is provided free by Gabonese Ministry of Health to public (and some private) ANC services. Free IPTp-SP is given orally to women during ANC visits by midwives and all pregnancy monitoring information (such as IPTp-SP) is reported in the health record. In total, 89.52% of pregnant women received at least one dose of IPTp-SP and 57.53 and 66.56% received at least three doses (complete IPTp-SP) during the first and the second sub-set of data, respectively. Since 2003, Gabon has adopted WHO recommendations [30] for ANC and led extensive awareness campaigns on the necessity of IPTp-SP. This study’s results show good adherence by pregnant women to this malaria control measure in southeastern Gabon, as is the case in other regions of the country. Studies led in Libreville and its surrounding areas, Lambaréné and Fougamou, have shown that complete IPTp-SP course, combined with adequate coverage of key interventions, good treatment-seeking behaviour and/or a low decline of chemoprevention efficacy, has led to a significant decrease in malaria prevalence in pregnant women [10, 12, 14, 15, 17, 31]. Similar results were noted in other African countries, such as Kenya and Burkina Faso [32, 33] although a low proportion IPTp-SP was reported in an urban area in the south of Benin in 2017 [24]. However, it should be noted that the proportion of women receiving at least three doses of IPTp-SP during their pregnancy has not changed since 2007 [10, 14]. The data support the fact that in urban centres, certain categories of women do not have access to socio-economic development. In Gabon, it is therefore urgent to explore new factors that may limit excellent coverage of IPTp-SP.

This study showed that pregnant women aged under 18 years old were those with the lowest complete IPTp-SP (Table 4). This could be explained by the fact that these women also attended ANC visits the least during their pregnancy (Table 2), given that prophylaxis with SP is administered during ANC visit by midwives. A study performed in Libreville showed that the complete IPTp-SP course was statistically linked to ANC visit attendance [10]. This study’s results are consistent with those of studies in the Democratic Republic of the Congo and in Burkina Faso, which showed that teenagers were less likely to attend ANC during pregnancy [26] and less likely to have complete IPTp-SP. These results could be due to several factors such as financial and geographical constraints, lack of knowledge of pregnancy risks and lack of education on the importance of ANC visit attendance. Low ANC visit attendance could also be due to socio-cultural aspects, such as the shame of being seen by relations, or beliefs that a pregnancy can be cursed or unsuccessful if it is revealed too early [34–36]. In contrast to pregnant women aged under 18 years, the ≥ 28 years group had the highest ANC visit attendance and the best complete IPTp-SP (Table 4). This could be explained by the fact that this age group want pregnancies due to marital status or age, and carefully seek and follow prenatal care.

This study showed that professional status of women impacted ANC visit attendance as well as complete IPTp-SP. Several Gabonese women declared that in the case of IPTp-SP, stock shortage in prenatal counselling services, women are often asked to buy their dose of IPTp-SP in drugstores, which is difficult for an underprivileged population. This was demonstrated in Burkina Faso in a study by Sinare-Ousmane in which 71% of pregnant women did not take enough doses of IPTp-SP because they were required to pay for treatment [37]. These observations are consistent with results obtained by Amani-Maleya et al*.* in the Democratic Republic of the Congo [26]. Furthermore, several studies have shown the importance of economic welfare and educational level for the adherence to IPTp-SP [26, 27, 38].

The mean weight of newborns did not differ significantly with that reported in Libreville between September 2005 and January 2006 after the implementation of IPTp-SP in Gabon [14]. This is not surprising as pregnant women are offered the same care in urban, semi-urban and rural regions in Gabon. The porportion of complete IPTp-SP in this study supports this result. Many studies have highlighted an increase in birth weight with the adoption of IPTp-SP [39–41]; birth weight was statistically linked with IPTp-SP course (Table 5). A significant birth weight gain for newborns was noted whose mothers took complete IPTp-SP course (Table 6). The data confirm those previously reported in Libreville and Lambaréné [12, 14] and in several countries of sub-Saharan Africa [42]. Despite the high prevalence of genotypes associated with resistance to SP in some rural areas in Gabon [31], the three-dose IPTp-SP policy must be maintained and improved in order to make it accessible to all pregnant women for effective malaria control. The proportion of LBW is higher in primipara than multipara (Table 7). This could be explained by the fact that young pregnant women are likely to be of lower parity (primipara) and that they women had low IPTp-SP course and can affect the health of their baby. The results are in contract to the meta-analysis which showed that the protective effect of IPTp-SP for pregnant women and the baby appeared to be limited to low parity women [43].

Malaria parasite-base diagnosis was performed by two microscopists. However molecular assay to detect many low-density infections was not used [44]*.* The prevalence of plasmodial infection in pregnant women was lower than those reported by Bouyou et al*.* in Libreville and by Mario Jäckle et al. in Fougamou (in the rural ares of Ngounié in Gabon) [14, 17]. The low prevalence observed could be a consequence of the adherence to IPTp-SP and treatment recommendations, or self-medication. No link was found between infection and prevention measures, such as LLINs, knowledge of malaria or window screens (Table 7). Furthermore, the prevalence of malaria in peripheral blood in pregnant women found in this study is lower than those reported in 1995 (25%), 2005 (12%) and 2011 (6%) [45]. However, parasitic sequestration and sub-microscopic infections support an underestimation of this prevalence. Indeed, a study showed that malaria diagnosis by polymerase chain reaction (PCR) leads to a better estimate of malaria prevalence after treatment with SP since the parasite load decreases in populations after the implementation of new disease control strategies [46]. These infections are a part of a plasmodial reservoir. In this study, *P. falciparum* infection was associated with LBW and premature births (Table 8). Similar results were found in Libreville [14] and in several other studies [42, 47]. The presence of parasites in the placenta disrupts exchanges between mother and fetus, limiting its development. No link was found between plasmodial infection, age and obstetric history (gravidity and parity) of pregnant women, unlike previous studies [15, 31, 48–50].

This study has a few limitations. There could be selection bias. Indeed, in the first sub-set study, data gathered on ANC care visit attendance and adherence to IPTp-SP were based on hospital birth registries and there were missing data. There could be selection bias (hospital-based), no molecular diagnosis had been carried out for *Plasmodium* infection, missing data and small data resulting in non-parametric distributions, and the absence of data on other outcomes associated with malaria infection in pregnancy such as stillbirths and spontaneous abortions. Also, data of several pregnant women were missing and could not be included in analyses. In addition, during data collection from January to April, 2020, several pregnant women were reluctant to answer questions, while others forgot some of their medical information. Finally, the parasitological examination of the umbilical cord and placenta could not be performed, especially as *P. falciparum* is often sequestered there, and placental infection is considered as one indicator of malaria in pregnant women.

## Conclusion

Several years after implementation of WHO and Gabonese Ministry of Health recommendations for pregnant women, this study showed that pregnant women attend ANC services. However, both complete ANC visit attendance and complete IPTp-SP course remains insufficient amongst women who give birth at CHRPMK despite high coverage of health insurance.

## Data Availability

Not applicable.

## Abbreviations

WHO: World Health Organization
LLINs: Long-lasting insecticidal nets
ANC: Antenatal care
IPTp-SP: Intermittent preventive treatment with sulfadoxine-pyrimethamine
CHRPMK: Centre Hospitalier Régional Paul Moukambi de Koula-Moutou
CIRMF: Centre Interdisciplinaire de Recherches Médicales de Franceville
LBW: Low birth weight
NHIF: National Health Insurance Fund
